# Integrated RNA and miRNA sequencing analysis reveals a complex regulatory network of *Magnolia sieboldii* seed germination

**DOI:** 10.1038/s41598-021-90270-y

**Published:** 2021-05-25

**Authors:** Mei Mei, Jun Wei, Wanfeng Ai, Lijie Zhang, Xiu-jun Lu

**Affiliations:** 1 Department of Horticulture, Shenyang Agricultural University, Shenyang, China; 2 Institute of Botany, Chinese Academy of Sciences, Beijing, China; 3 Department of Forestry, Shenyang Agricultural University, Shenyang, China

**Keywords:** Transcriptomics, Molecular biology, Transcriptomics, Plant sciences, Plant hormones

## Abstract

*Magnolia sieboldii* K. Koch (*M. sieboldii*) is a deciduous Chinese tree species of the *Magnoliaceae* family with high ornamental, medicinal, and economic benefits. The germination of *M. sieboldii* seeds under natural conditions is extremely difficult, thereby hindering the cultivation and breeding of this important species. The molecular mechanisms underlying *M. sieboldii* seed germination remain unclear due to the lack of genomic and transcriptomic resources. Here, we integrated both mRNA and miRNA sequencing to identify the genes and pathways related to *M. sieboldii* germination. A comprehensive full-length transcriptome containing 158,083 high-quality unigenes was obtained by single-molecule real-time (SMRT) sequencing technology. We identified a total of 13,877 genes that were differentially expressed between non-germinated and germinated seeds. These genes were mainly involved in plant hormone signal transduction and diverse metabolic pathways such as those involving lipids, sugars, and amino acids. Our results also identified a complex regulatory network between miRNAs and their target genes. Taken together, we present the first transcriptome of *M. sieboldii* and provide key genes and pathways associated with seed germination for further characterization. Future studies of the molecular basis of seed germination will facilitate the genetic improvement *M. sieboldii*.

## Introduction

*Magnolia sieboldii* K. Koch is a deciduous Chinese tree species of the *Magnoliaceae* family with high ornamental, medicinal, and economic benefits^1,2^. Extracts from *M. sieboldii* plants are used in medicine due to their excellent inhibitory effects on tyrosinase activity and melanin production. The timber of the *M. sieboldii* tree can be used to make furniture. Presently, the wild resource of this species has been jeopardized due to unreasonable exploitation and utilization. In addition, *M. sieboldii* seeds are difficult to germinate even after stimulation by a long period of cold stratification, making the cultivation and breeding of this species very challenging^3^. While the germination of *M. sieboldii* seeds has been investigated by physiological^4–6^ methods and proteomics^7,8^ analyses, the molecular mechanisms underlying its seed dormancy and germination still remain largely unknown.

Different plant species have developed different dormancy strategies to regulate the timing of seed germination, such as morphological dormancy (MD), physiological dormancy (PD), and morphophysiological dormancy (MPD). In MD, the embryo is not fully developed and must further develop before germination can occur. In PD, embryo growth and seed germination are inhibited until inhibitory chemicals are eliminated, which is often induced in cool and moist conditions. *M. sieboldii* seeds exhibit MPD, that is, a combination of MD and PD^9^. A study has reported that mature *M. sieboldii* seeds contain under-developed embryos and large endosperms^10^. Additionally, constraints caused by the seed coat are a major factor associating with MPD in *M. sieboldii* seeds. For example, the external seed coat primarily inhibits the development of cotyledons and radicles^6^. *M. sieboldii* seeds usually germinate with a low rate and irregular shape, which requires a long germination period (150 days) before germination under natural conditions^3^. Therefore, *M. sieboldii* seeds must undergo embryo growth to release MD and exposure to controlled environmental conditions (temperature and moisture) to stimulate PD before entering germination^11^. This makes the germination process of MPD seeds more complicated compared to either PD or MD seeds.

Seed dormancy and germination are complex processes, and previous studies have demonstrated that multiple metabolic pathways are involved. These pathways include hormone signaling to release dormancy^12,13^, lipid and starch metabolism to provide energy^14^, osmotic regulation of protein hikes to eliminate germination inhibitors^15^, as well as other processes. Abscisic acid (ABA) and gibberellin acid (GA) have been identified as the primary endogenous factors regulating the transition from dormancy to germination. Moreover, various genes with different functions have been identified and demonstrated to show differential expression between dormant and germinated seeds. For example, the *DELAY OF GERMINATION1* (*DOG1*) gene is a key regulator of seed germination, acting as a timer for seed dormancy release in *Arabidopsis*^16^. Genetic studies have also suggested that a network of transcriptional regulators plays an important role in seed germination. In *Arabidopsis*, various transcription factors have been identified to function as positive regulators of seed germination. For example, *LEAFY COTYLEDON1* (*LEC1*) encodes a CCAAT-box binding protein that promotes the expression of *DOG1*^17^. On the other hand, RNA silencing is an important mechanism of gene regulation, and alterations in the levels of microRNAs affect seed dormancy and germination. For example, miRNA159 is involved in ABA/GA signaling pathways during seed germination in *Arabidopsis*^18^. In wheat, the overexpression of miR9678 delayed germination, and its knockdown enhanced the germination rate^19^. These findings highlighted that seed dormancy and germination is governed by the expression and regulation of a complex network of genes. Additionally, it demonstrated that genome-wide differential gene expression analysis between non-germinated and germinated seeds is an effective way to identify the key genes and pathways involved in seed germination. However, almost no genomic or transcriptomic data are available for *M. sieboldii*.

RNA sequencing (RNA-seq) has become a powerful tool for the transcriptome-wide analysis of differentially expressed genes (DEGs) within the regulatory network^20–22^. RNA-seq via second-generation sequencing technology produces both qualitative (transcript sequences) and quantitative (transcript levels) data with high level of sensitivity. However, short reads require large computational assemblies and cannot span full-length transcripts, which reduces the accuracy of transcript prediction. Recently, single-molecule real-time (SMRT) sequencing from Pacific Biosciences (PacBio) has been reported to generate long reads^23–25^, thereby providing an efficient approach to sequencing full-length cDNAs without further assembly. SMRT sequencing is highly suitable for isoform discovery in de novo transcriptomic analysis^26^. Presently, an effective approach to understanding the gene expression network in non-model species is to combine both SMRT sequencing and RNA sequencing methods^27,28^.

In the present study, we applied SMRT sequencing to generate the full-length transcriptome for *M. sieboldii*. To understand the molecular mechanisms underlying seed germination in *M. sieboldii*, we identified DEGs between non-germinated and germinated seeds and analyzed their biological functions in non-germinated and germinated seeds. We also combined RNA and miRNA sequencing to analyze the interactions between miRNAs and their target genes. To our knowledge, this is the first report on the transcriptome profiling of *M. sieboldii* seed. This study provides a comprehensive understanding of seed germination in *M. sieboldii*.

## Results

### Construction and annotation of the full-length transcriptome for *M. sieboldii*

To identify as many transcripts as possible, we collected high-quality RNA samples from seven different developmental stages during seed germination for the construction of the full-length transcriptome. The non-germinated and germinated seeds are presented in Supplementary Fig. S1 online. The non-germinated seeds were sampled before stratification. The germinated seed were sampled 90 days after stratification when radicles grew to a length of 2–3 mm and broke through the seed coat. After quality control, a total of 1,170,345 circular consensus sequences (CCS) were obtained from two SMRT cells with a total length of 2,967,247,371 bases. The average length of CCS reads was 2,535 bases. All CCS reads were classified into 708,510 full-length non-chimeric sequences and 436,360 non-full-length sequences. These consensus sequences were further clustered and polished to yield 403,260 full-length consensus isoforms, including 51,561 polished high-quality and 348,353 low-quality transcripts. After removing redundant sequences, we finally obtained 158,083 unigenes with a mean length of 3,003 bp and N50 of 3,910 bp.

A total of 131,796 unigenes (83.4%) were annotated by searching public protein databases (see Supplementary Fig. S2a online). Among these annotated unigenes, 131,289 and 97,433 transcripts were found to have homologs in Nr and Swiss-Prot databases, respectively. The three species showing the most hits in the NCBI non-redundant protein database were *Nelumbo nucifera*, *Macleaya cordata*, and *Vitis vinifera*. A total of 26,287 transcripts did not return any matches and, therefore, they were considered as potentially novel genes in *M. sieboldii*. Furthermore, 72,978 and 28,365 transcripts were assigned to different functional terms in three GO categories and KEGG pathways, respectively. For these GO categories, the functional terms ‘cellular process’ and ‘metabolic process’ were the predominant annotations in the biological process category. In the cellular component category, ‘cell part’ and ‘organelle’ were the two most abundant functional terms. In the molecular function category, ‘binding’ and ‘catalytic activity’ were the two most common functional terms (see Supplementary Fig. S2b online). For the KEGG pathway annotations, the most annotated genes were associated with either ‘metabolism’ or ‘genetic information processing’. In the metabolism category, ‘carbohydrate’, ‘amino acid’, and ‘lipid’ were the three most abundant metabolic pathways. In the genetic information processing category, ‘translation’, ‘degradation’, and ‘transcription’ were the three most abundant pathways. (see Supplementary Fig. S2c online). In addition, ‘post-translational modification’ and ‘signal transduction’ among the COG function classification were associated with the most annotated gene number, except for the general function predictions (see Supplementary Fig. S2d online). These results show that SMRT sequencing provided a large number of high-quality full-length transcripts in *M. sieboldii* and most of the transcripts were annotated using the public databases.

### DEGs between non-germinated and germinated seeds

**Figure 1:**
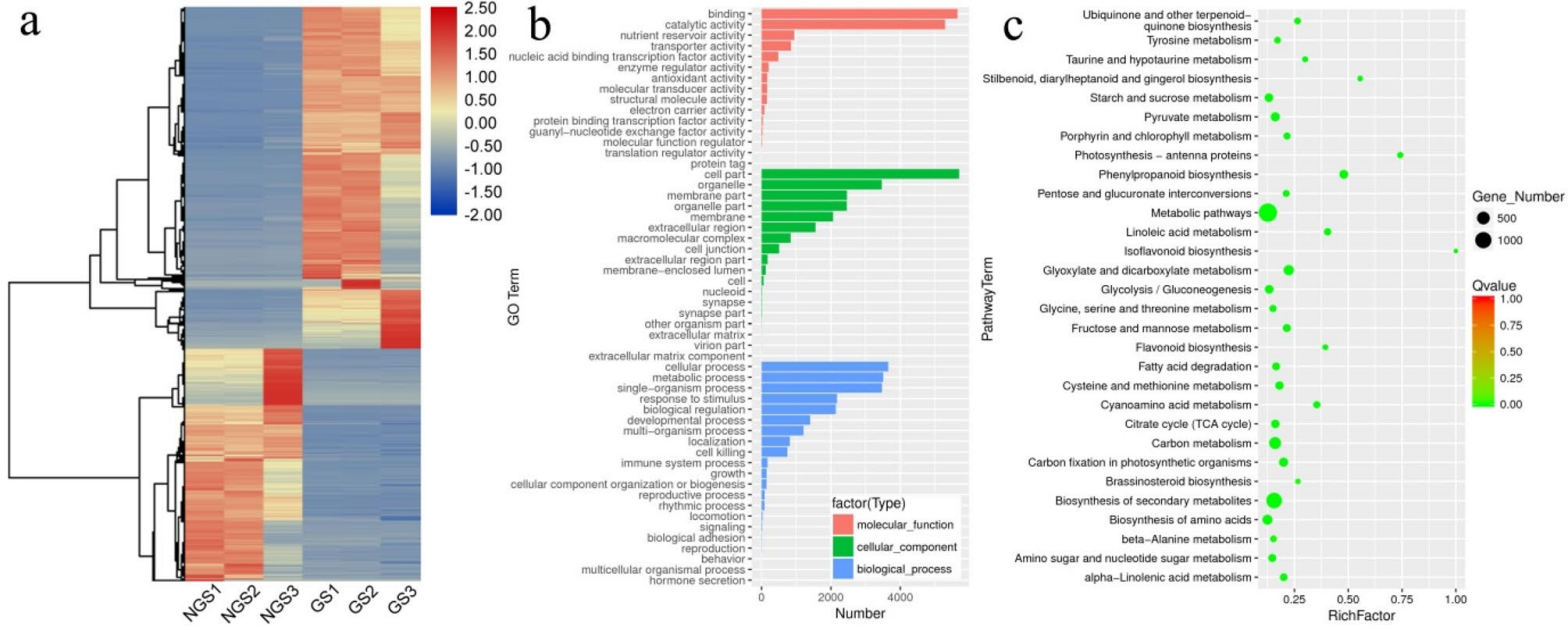
Differentially expressed genes (DEGs) between non-germinated and germinated seeds. (**a**) Hierarchical clustering graph of 13,877 DEGs based on log2FPKM values. Red and blue indicate transcripts with increased and decreased levels, respectively. *NGS* non-germinated seeds, *GS* germinated seeds. (**b**) GO enrichment analysis based on DEGs between non-germinated and germinated seeds. Vertical coordinates indicate the secondary classification of gene ontology; horizontal coordinates indicate the number of related genes. Different colors indicate the three categories of the gene ontology terms. (**c**) KEGG pathway enrichment analysis based on DEGs between non-germinated and germinated seeds. The vertical axis represents the pathway identity; the horizontal axis represents the rich factor. The size of the circle represents the number of differentially expressed genes in this pathway, and the color of the circle corresponds to the significance level of the enrichment.

To identify DEGs between non-germinated and germinated seeds, RNA-seq clean reads were mapped to the reference transcriptome generated by SMRT sequencing. The mapping rate ranged from 87 to 93% among different libraries and the mapped read-count for each transcript was converted into the expected number of fragments per kilobase of transcript per million base pairs (FPKM). Differential analysis using DESeq2 revealed that a total of 13,877 transcripts were differentially expressed, including 7898 up-regulated and 5,979 down-regulated transcripts between non-germinated and germinated seeds. Hierarchical clustering of all DEGs revealed that gene expression profiles were similar between replicated samples but significantly different between non-germinated and germinated seeds samples. Furthermore, clustering also classified all DEGs into two sub-clusters. The first sub-cluster contained genes that were up-regulated in the non-germinated seeds but down-regulated in germinated seeds. The second sub-cluster contained genes that were down-regulated in the non-germinated seeds but up-regulated in germinated seeds (Fig. 1a).

We used GO and KEGG enrichment analyses to characterize the functions of the DEGs. (Fig. 1b). Of the assigned GO terms, the molecular functions were mainly related to ‘binding and catalytic activity’. Significantly enriched GO terms for the cellular components were almost all related to ‘general cellular regions’ such as cell parts and organelles. For the biological process category, the six most enriched GO terms were associated with ‘cellular process’, ‘metabolic process’, ‘single-organism process’, ‘response to stimulus’, ‘biological regulation’, and ‘developmental process’. Furthermore, the KEGG enrichment results revealed that metabolic pathways play an important role in seed germination (Fig. 1c). Of the genes associated with the significantly enriched KEGG pathways, those involved in starch and sucrose metabolism, fatty acid degradation, photosynthesis, and plant hormone biosynthesis were the most probable candidates related to seed germination in *M. sieboldii*.

### DEGs related to plant hormones signal transduction for seed germination

**Figure 2:**
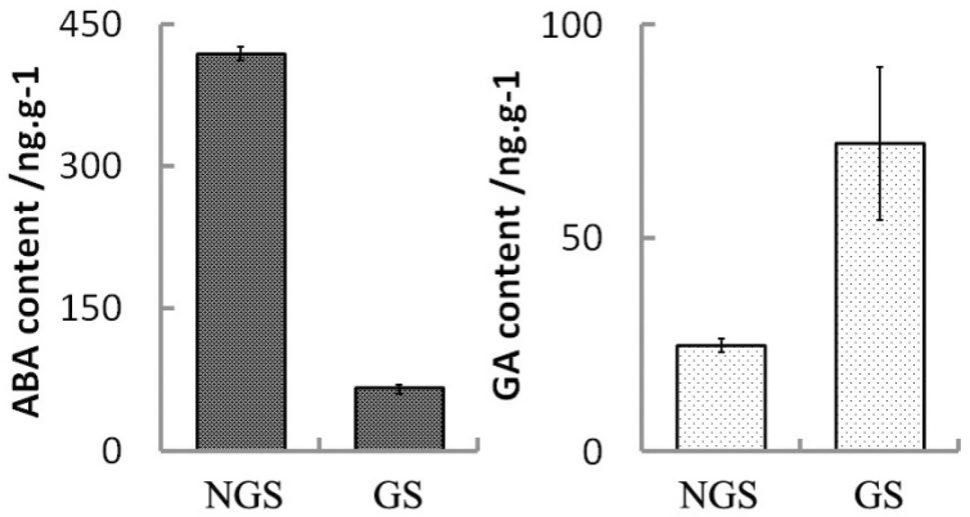
Measurement of ABA and GA_3_ levels in non-germinated and germinated seeds. *NGS* non-germinated seeds; *GS* germinated seeds.

**Figure 3:**
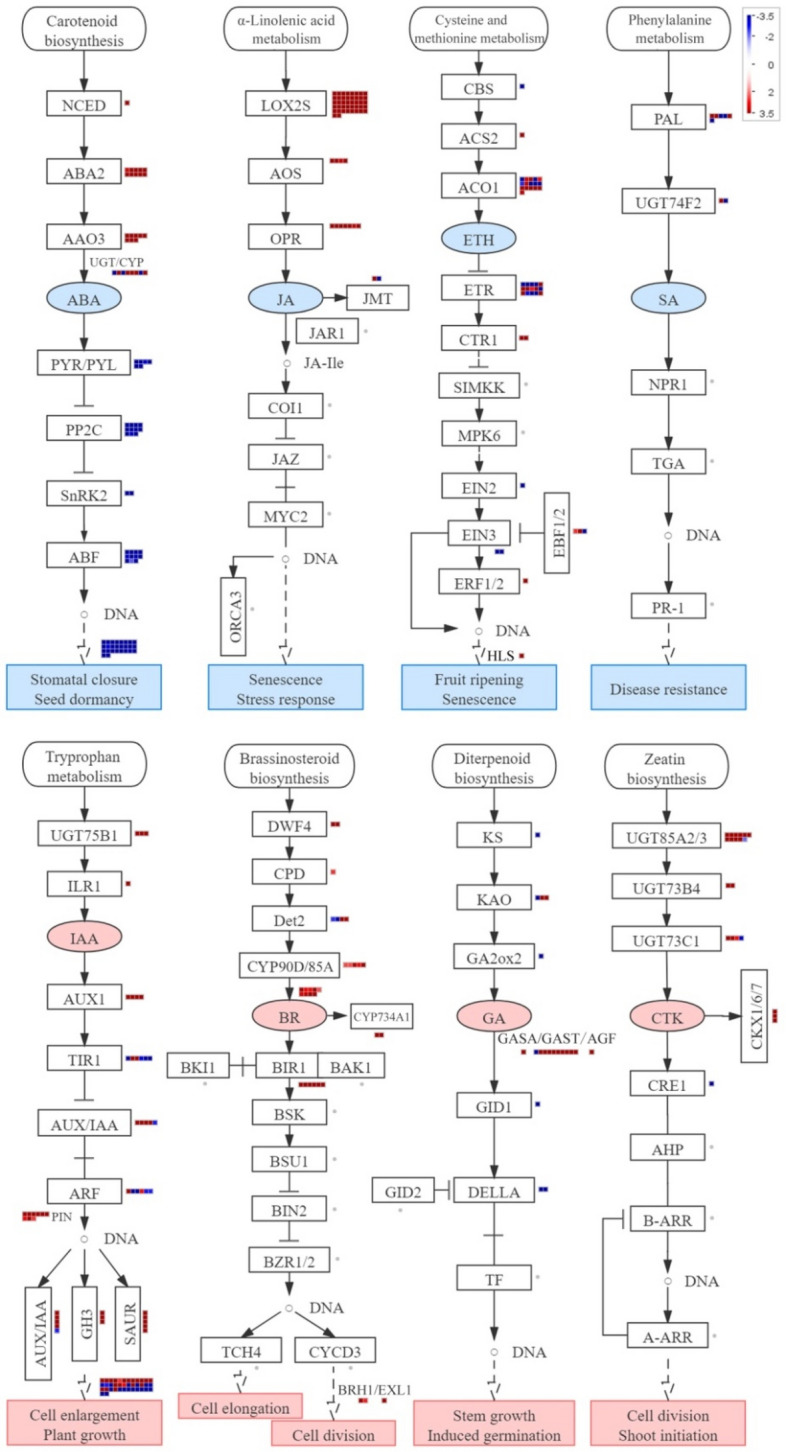
Schematic of DEGs involved in plant hormone pathways^34^. Key enzymes and proteins are presented as their names. Red dots indicate up-regulated genes; blue dots indicate down-regulated genes.

Plant hormones have been demonstrated to play important roles in seed dormancy and germination. We measured and compared ABA and GA_3_ levels in non-germinated and germinated seeds. The ABA content was significantly decreased, while the GA_3_ content was significantly increased in germinated seeds (Fig. 2). This observation confirmed the key role of ABA and GA_3_ in seed germination. We also analyzed the DEGs involved in various plant hormone regulation pathways (Fig. 3). A total of 27 DEGs were related to ABA metabolism, and almost all the genes were up-regulated, which is in agreement with the low content of ABA in germinated seeds. For example, *CYP707A2* that encodes ABA 8’-hydroxylase (a key enzyme in ABA catabolism) was up-regulated in germinated seeds. ABA 8’-hydroxylation is thought to play an important role in ABA catabolism and *CYP707A2* has been demonstrated to be responsible for the rapid decrease of ABA level during seed imbibition^29^. A total of 14 DEGs were involved in ABA signal transduction, and all were down-regulated, including the *ABI3* and *LEC2* genes. The *ABI3* gene is known to play an important role during seed maturation and dormancy, which has been involved in not only in the ABA signaling cascade but also to adjust development in case of environmental stress^30^. The *LEC2*, a B_3_ DNA binding domain transcription factor, regulates seed storage protein and oil biosynthesis and has a central regulatory role in embryo development and seed maturation in soybean^31^. The *Arabidopsis abi3* mutants show reduced response to ABA and *LEC2* plays an important role in the suppression of premature germination. A total of 20 DEGs were matched in the GA_3_ metabolism pathway or signal transduction, and the genes involved in the degradation pathway of GA_3_ were all down-regulated. We found that *ent-kaurenoic acid oxidase* (*CYP88A*) was involved in the seed germination process, which catalyzes three steps of the gibberellin biosynthesis pathways^32^. We also observed the gene expression change of two DELLA proteins in GA signal transduction, which has been demonstrated to control seed germination in *Arabidopsis*^33^. Only four DEGs were matched in the biosynthesis of IAA, and all were up-regulated, while 23 DEGs were matched in the signal transduction of IAA, and most were up-regulated. Thromboxane-A synthase, a member of the CYP711A cytochrome P450 family that controls meristem formation, was significantly up-regulated. A total of 38 up-regulated DEGs were annotated as auxin response factors, which promote cell elongation and seed germination. In addition, 31, 21, 56, and 18 DEGs were matched in the BR, CTK, JA, and ETH pathways, respectively. Among them, BR6OX2 was up-regulated and involved in the biosynthesis of BR. All DEGs in JA metabolism were up-regulated, except the encoding cystathionine beta-synthase, which was down-regulated. These results suggest that a complex regulatory network associates with multiple plant hormones during seed germination.

### DEGs related to various metabolisms for seed germination

**Figure 4:**
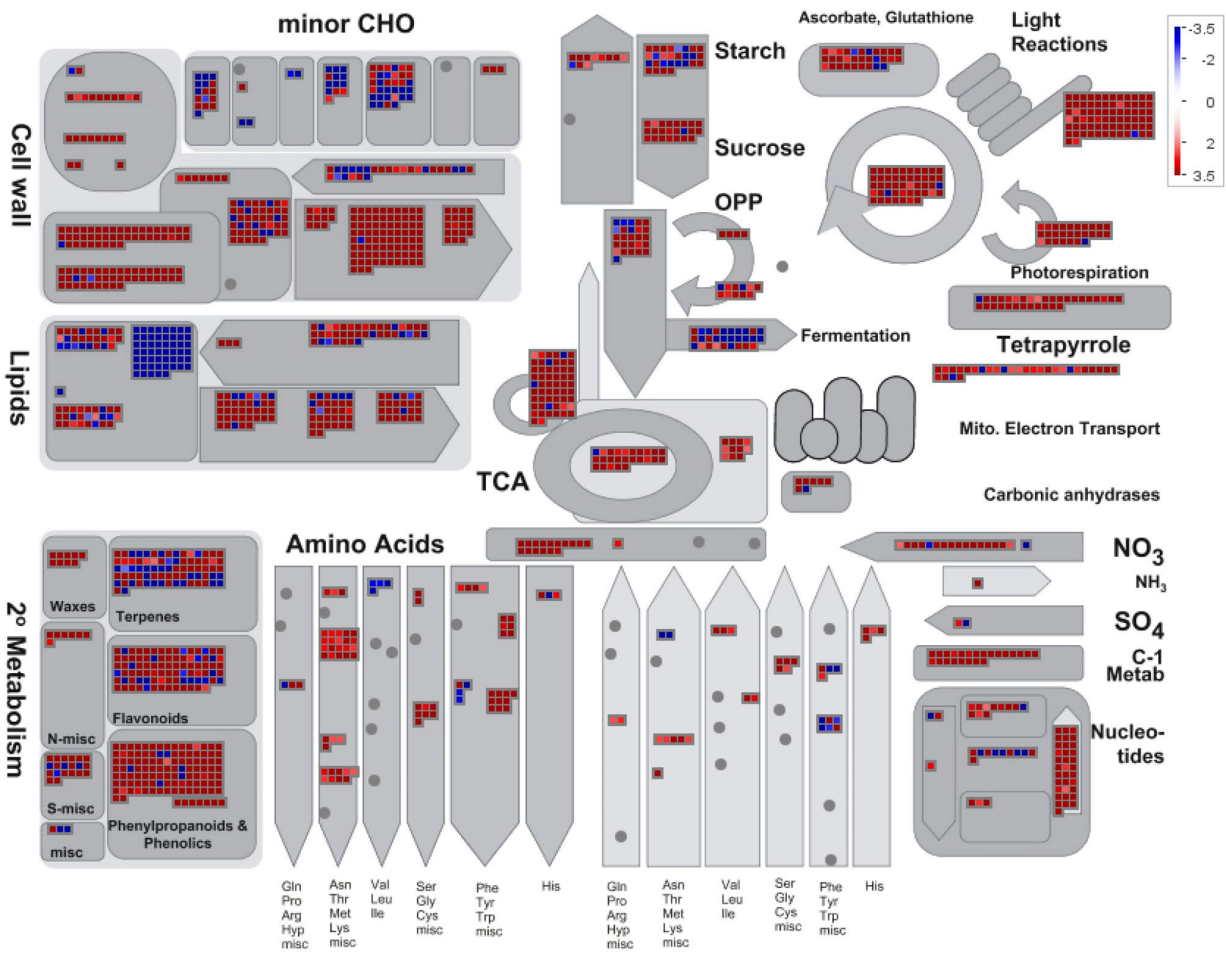
Overview of DEGs associated with diverse pathways. Red and blue rectangles indicate up-regulated and down-regulated genes, respectively.

Given the importance of metabolic pathways during seed germination revealed by GO and KEGG enrichment analyses, we overviewed the 10,028 DEGs involved in various metabolic pathways in Fig. 4. The most abundant DEGs were related to cell wall metabolism, and most were up-regulated in germinated seeds and involved in cell wall degradation and modification. Among them, genes encoding pectinesterases and expansions were the most significantly up-regulated genes. We also observed numerous DEGs that were related to the metabolism of lipids, starches, and amino acids, which contributed to energy production for seed germination via the tricarboxylic acid (TCA) cycle. For example, we identified DEGs related to starch and sucrose metabolism in the energy production process of fermentation. Additionally, a large number of triacylglycerol synthesis genes were down-regulated, which impeded triacylglycerol synthesis. This step may be important for the germination of seeds with high oil content such as *M. sieboldii*. We identified 23 differentially expressed light-harvesting chlorophyll protein complex (*LHC*) genes that related to photosynthesis. All *LHC* genes were up-regulated in germinated seeds, except for *LHCA5* and *LHCB7*. The remaining DEGs were mainly related to secondary metabolic processes, which involved terpenes, flavonoids, and phenylpropanoids. According to GO and KEGG enrichment analyses, four metabolic pathways, including those of flavonoid biosynthesis, phenylpropanoid biosynthesis, isflavonoid biosynthesis, and stilbenoid, diarylheptanoid and gingerol biosynthesis, were closely connected and found to have a high rich factor based on KEGG enrichment analysis (see Supplementary Fig. S3 online). The results indicated that the *PAL*, *4CL*, and *CYP73A* genes were up-regulated to convent phenylalanine to p-coumaroyl-CoA. In addition, 38 *PER42* genes were up-regulated and 25 peroxiredoxin 6 genes (*PRDX6*) were down-regulated. These genes were involved in the response to stress by detoxifying peroxides, which is probably associated with the initial seed imbibition (uptake of water) and cold condition. These results suggest that energy production, photosynthesis, and stress-related metabolic pathways may be involved in the germination process of *M. sieboldii* seeds.

### Expression dynamics of miRNAs and their target genes in seed germination

**Figure 5:**
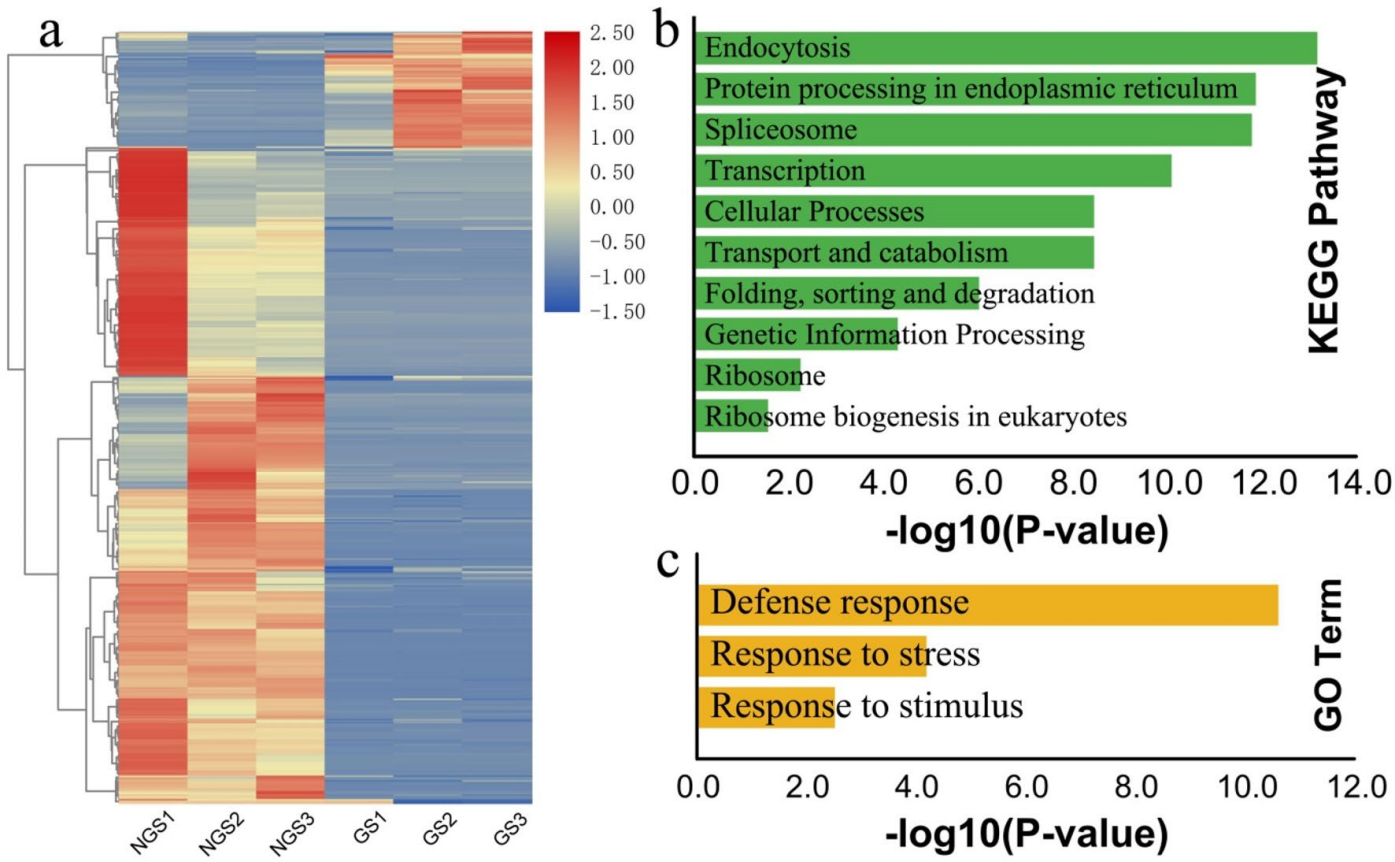
Hierarchical clustering and GO/KEGG enrichment analyses for the target genes of the differentially expressed miRNAs. (**a**) Hierarchical clustering of target genes based on log_2_FPKM values. Red and blue indicate transcripts with increased and decreased levels, respectively. *NGS* non-germinated seeds, *GS* germinated seeds. (**b**) KEGG pathway enrichment analysis and (**c**) GO enrichment analysis.

**Figure 6:**
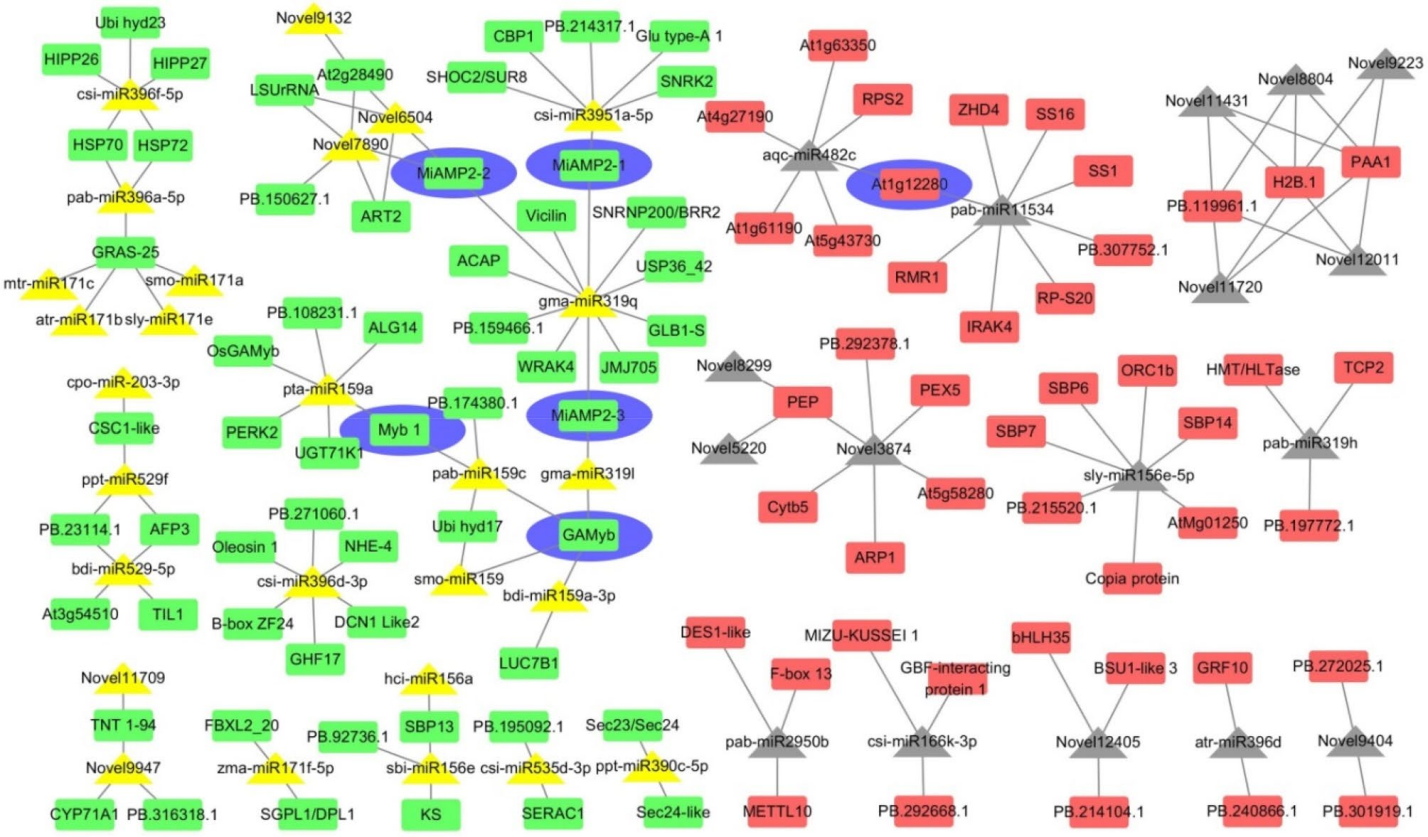
miRNA–mRNA interaction network. The miRNA–mRNA interaction network map was generated by Cytoscape (v3.6.0). Triangles represent miRNAs; rectangles represent their target genes. The hub genes of the interaction network are highlighted in blue. Yellow and gray indicate up-regulated and down-regulated miRNAs, respectively. Red and green indicate up-regulated and down-regulated target genes, respectively.

A total of 68 differentially expressed miRNAs were identified, including 40 up-regulated miRNAs and 28 down-regulated miRNAs (Fig. 5a). According to the prediction from TargetFinder Software and the negative correlations of gene expression levels between miRNAs and mRNAs (target genes), a total of 490 target genes were identified. Among these target genes, 42 and 130 genes were annotated in GO and KEGG databases, respectively. KEGG enrichment analysis indicated that the target genes were mainly associated with ‘endocytosis’, ‘protein process’, ‘spliceosome’, ‘transcription’, and ‘cellular process’ (Fig. 5b). In GO enrichment analysis, target genes were found in three GO terms, including ‘defense response’, ‘response to stress’, and ‘response to stimulus’ (Fig. 5c). We then constructed the interaction network between differentially expressed miRNAs and their target genes, which resulted in 571 miRNA–mRNA pairs (Fig. 6). For example, *miRNA159* targets the transcription factor *GAMyb*, which functions with *MYBS1* to integrate diverse nutrient starvation and GA signaling pathways during germination. miRNA159 also targets genes encoding UDP-glycosyltransferase 71K1 (*UGT71K1*), proline-rich receptor-like protein kinase PERK2 (*PERX2*), and UDP-N-acetylglucosamine transferase subunit ALG14 homolog (*ALG14*), which are key enzymes in sugar metabolism. In addition, *miR319* targets multiple members of the cupin superfamily, which includes those encoding various seed storage proteins, including globutin-1, GTPase-activating protein, and vicilin-like antimicrobial peptides (*MiAMP2-1*). *miRNA395* targets the CCG-binding protein 1 (*CBP1*), glutelin type-A1, and *MiAMP2-1* genes. Interestingly, *MiAMP2-1* is regulated by both *miR319* and *miRNA395*. *CBP1* is required for embryo development, and glutelin is an important seed storage protein. These results indicate that miRNAs were involved in multiple aspects of seed germination such as sugar and storage protein metabolism and plant hormone signal transduction. The interaction network also provides a starting point for revealing the complex regulatory mechanism of seed germination in *M. sieboldii*.

## Discussion

Currently, there are almost no genomic and transcriptomic resources in *M. sieboldii*. The lack of genomic and transcriptomic resources has been a major barrier to understanding the functions and regulations of genes involved in plant biological processes. In the past, short-read RNA-seq has become an effective tool to evaluate whole mRNA expression patterns in many species^35–37^. However, short-read RNA-seq is insufficient in constructing an accurate and a reliable transcriptome for gene expression estimation, especially for species without reference genomes. SMRT sequencing from PacBio can generate full-length transcripts without assembly^38–40^. In this study, we constructed a full-length transcriptome using PacBio Iso-seq and investigated the gene expression pattern using short-read RNA-seq. This experimental design resulted in high-quality transcript identification and accurate gene expression estimation. For example, > 90% of RNA-seq reads could be aligned with the reference transcriptome. In addition, we found a high degree of correlation for gene expression between different biological replicates. Moreover, we successfully identified key genes and pathways involved in seed germination based on our experimental design. These results demonstrated that combining SMRT sequencing and RNA-seq is a powerful approach in transcriptome profiling for non-model species such as *M. sieboldii.*

Plant hormones are internal mediators of plant development and environmental factors^41–43^. Our results support a potential role of plant hormones in *M. sieboldii* seed germination. Firstly, changes in ABA and GA_3_ levels were observed during seed germination, and germinated seeds presented a lower ABA level but a higher GA_3_ level compared with non-germinated seeds. This observation is consistent with the roles of ABA and GA in seed germination. ABA reversibly arrests embryo development upon radicle growth initiation^44^. GA has been reported to improve seed germination in multiple species^45,46^. Secondly, we identified multiple genes that were involved in ABA and GA signal transduction such as the *ABI3* and *DELLA* genes. The *abi3* mutation in *Arabidopsis* confers the reduced response to ABA^47^, and DELLA protein is a key negative regulator of the GA signaling pathway^48^. Thirdly, we identified DEGs in other plant hormone pathways besides ABA and GA, including auxin, brassinosteroid, jasmonic acid, cytokinin, and ethylene. For example, the gene that encodes thromboxane-A synthase, a member of the *CYP711A* cytochrome P450 family that controls the formation of the meristem via the auxin signaling pathway, was significantly up-regulated^49^. Taken together, our results indicate that plant hormones are involved in *M. sieboldii* seed germination through individual signaling pathway and complex signaling networks.

All seeds need water, oxygen, proper temperature, and sufficient energy in order to germinate. Our results suggest that energy production-, photosynthesis-, and stress-related metabolic pathways play important roles in seed germination in *M. sieboldii*. Numerous DEGs identified in this study were related to the metabolism of lipids, starches, and amino acids, which probably contributed to energy production. Interestingly, lipid synthesis genes were up-regulated in germinated seeds, as *M. sieboldii* seeds have a high oil content (39%)^1,50^. An oleoside protein was found to be associated with lipid accumulation in seeds, and its knockdown resulted in an abnormal embryo phenotype during seed germination, indicating that the maintenance of a high level of oil content is essential during *M. sieboldii* seed germination. Recently, increasing evidence has revealed that amino acids are not only used for the synthesis of storage proteins, they can also be catabolized, and their catabolic products can feed into the TCA cycle to generate energy^51^. During seed germination, there is limited light and oxygen for photosynthesis so most photosynthesis-related genes are silent. In this study, we identified 23 differentially expressed light-harvesting chlorophyll protein complex (*LHC*) genes, and almost all *LHCs* were up-regulated in germinated compared with non-germinated seeds. This observation suggests that photosynthesis plays an important role after cotyledons have emerged through the seed coat, which probably contributed a considerable amount of oxygen to seeds. Abiotic stress can affect seed germination, but plants have developed unique strategies to ensure germination^52,53^. For example, many DEGs were involved in the response to stress by detoxifying peroxides, which is probably associated with the initial seed imbibition (uptake of water) and cold conditions. These results indicate that multiple metabolic pathways were involved during seed germination to ensure sufficient energy production and stress responses in addition to plant hormone signal transduction.

Previous studies have demonstrated that plant miRNAs regulate gene expression during seed germination^54,55^. In this study, a comprehensive vision was developed to understand the functions of miRNAs during seed germination with integrated data from both mRNA and miRNA sequencing. Our results revealed a correlation between miRNAs and the expression of genes involved in sugar and amino acid metabolism and plant hormone signal transduction during *M. sieboldii* seed germination. It is interesting that the target genes of differentially expressed miRNAs identified were consistent with the DEGs identified by mRNA-seq analysis, which implies the robustness of our experimental design. We also observed a complex regulatory network between miRNAs and their targets in controlling seed germination. A total of 68 differentially expressed miRNAs and 490 target genes were identified. Among these interacting pairs, we noticed that a single miRNA could target multiple genes, and sometimes two different miRNAs could target a single gene, such as *MiAMP2-1*. The complex regulatory crosstalk between miRNAs and plant hormones was also confirmed in this study. For example, *miRNA159* targets the transcription factor *GAMyb*, which functions with *MYBS1* to integrate diverse nutrient starvation and GA signaling pathways during germination^56,57^. miRNA159 is known to control both the activation and repression of seed germination and dormancy^18,58^. In addition, we identified unknown miRNAs in this study, although their functions and regulatory networks remain largely unknown. Further functional studies, such as those involving transgenic and bioinformatic analyses, could help to shed light on these unknown miRNAs.

In this study, we integrated SMRT sequencing, RNA-seq, and miRNA-seq to construct a comprehensive transcriptome of *M. sieboldii* seed and to obtain an overview of the transcriptomic landscape during *M. sieboldii* seed germination. To our knowledge, this is the first transcriptome profiling report in *M. sieboldii.* The results of this study also provided a global view of mRNA and miRNA regulation during seed germination, and the key genes identified, which were differentially expressed between non-germinated and germinated seeds, provided the targets for the further characterization of the molecular mechanisms underlying seed germination in *M. sieboldii.*

## Materials and methods

### Plant materials and RNA extraction

*M. sieboldii* seeds were harvested in Benxi, Liaoning Province, China (40_490N, 123_340E) in October 2015, and two kilograms of seeds were used in this study. To obtain germinated seed samples, all seeds were mixed with moist sand and maintained under controlled conditions (45 days at 0 °C–5 °C, 15 days at -10 °C–10 °C, and 30 days at 15 °C–18 °C) in 2016. The conditions used have been confirmed to satisfy the seed germination requirements based on a previous study of our lab^5^. Seeds at seven prolonged germination time points (0, 15, 30, 45, 60, 75, and 90 days after starting the stratification) were collected, snap frozen in liquid nitrogen, and stored at − 80 °C for RNA extraction. Seeds at the first time point (before stratification) were defined as non-germinated seeds (NGS). Seeds at the last time point (90 days) were defined as germinated seeds (GS) when radicles grew to a length of 2–3 mm and broke through the seed coat (see Supplementary Fig. S1 online). Total RNA from seeds at each time point was extracted using the RNAprep Pure Plant Kit from TIANGEN Biotech Co., Ltd. (Beijing, China) according to the manufacturer’s instructions and digested with RNase-free DNase (Manufacture’s info) to remove genomic DNA. The quality of RNA was checked using the NanoDrop Spectrophotometer (Thermo Fisher Scientific Inc.) and Agilent 2100 Bioanalyzer (Agilent Technologies, Palo Alto, CA, USA). Only extractions with an RNA integrity value greater than 9 were used further.

### PacBio Iso-seq library preparation and sequencing

To obtain the comprehensive full-length transcriptome, equal amount of the RNA samples from the seven time points were pooled and used for Iso-seq library preparation. First stand cDNA synthesis was performed using the SMARTer PCR cDNA Synthesis Kit (Clontech) and subsequently used for large-scale PCR to generate double-stranded cDNA by PrimeSTAR GXL DNA Polymerase (Clontech) according to the manufacturers’ instructions. The amplified cDNA was then cleaned up by Ampure PB Beads, and full-length transcripts with sizes up to 4 kb were collected. At the same time, < 4 kb transcripts were collected using the BluePippin Size-Selection System (Sage Science, Inc., MA, USA) to enrich the transcript concentration. Both < 4 kb and > 4 kb transcripts were separately used for SMRTbell library construction using the PacBio Template Prep Kit (Pacific Biosciences of California, Inc., California, USA). The established libraries were validated using the Agilent 2100 Bioanalyzer and quantified using the Qubit 2.0 Fluorometer (Invitrogen, Carlsbad, CA, USA). Finally, two SMRT cells were run on the PacBio Sequel System using P6-C4 chemistry.

### Full-length transcriptome analysis pipeline

SMRT Link 4.0 Software (https://github.com/PacificBiosciences/SMRT-Link) and the Iso-seq pipeline were used to process the raw data. Briefly, the circular consensus sequence (CCS) was extracted from raw subreads produced from the PacBio Sequel System. CCSs were then classified into full-length non-chimeric (FLNC) and non-full-length (NFL) reads based on the presence of the poly(A) tail as well as 5′ and 3′ cDNA primers. FLNC reads were clustered into consensus sequences using the Iterative Clustering for Error Correction (ICE) algorithm. Combined with NFL reads, consensus sequences were polished using the Arrow Program (https://github.com/PacificBiosciences/gcpp). The resulting consensus sequences were divided into high-quality and low-quality sequences based on the post-correction accuracy criterion (> 0.99). To further improve the accuracy of transcripts, low-quality consensus sequences were corrected by Illumina short reads using CoLoRmap Software^59^ with default parameters. The corrected low-quality and high-quality isoforms were combined as high-quality full-length transcripts. Finally, the CD-HIT-EST (http://weizhongli-lab.org/cd-hit/) Program was used to remove redundant sequences to obtain final high-quality transcripts, which were considered as the reference transcriptome and used for further analysis.

### Transcript structure prediction and functional annotation

Putative genes and their protein-coding regions were predicted using TransDecoder (v3.0.0) Software. To classify the alternative splicing events, AStalavista Software (v4.0) was used to identify five major alternative splicing types, including intron retentions, exon skips, alternative 3′-acceptors, alternative 5′-donors, and mutually exclusive exons. This software takes a GTF file produced by the GffCompare Package as its input. For novel transcript prediction, each isoform was compared with existing gene models by the Cuffcompare Program, and the isoforms were further classified into nine groups based on their exon structures (splicing junctions). Thereafter, novel transcripts could be predicted using GffCompare Program. To identify fusion transcripts, the criteria used for a single transcript were as follows: (a) FL transcripts mapping to two or more loci; (b) each mapped locus aligning with at least 5% of the transcript; (c) a combined alignment coverage of at least 99%; and (d) a distance of at least 100 kb between mapped loci. Functional annotation was performed using BLAST to compare transcripts with Nr, Swiss-Prot, COG, GO, and KEGG databases with an E-value threshold of 1e-5. Only the best blast hit was reserved for gene annotation.

### mRNA sequencing and data analysis pipeline

Seeds from three biological replicates were sampled, and only RNA samples from the first (NGS, day 0) and last (GS, day 90) time points were used for mRNA sequencing. A total of six mRNA-seq libraries was constructed using the NEBNext Ultra RNA Library Prep Kit following the manufacturer’s instructions. Libraries were then sequenced on the Illumina Hi-seq Platform (Illumina, San Diego, CA, USA) to generate 150 bp paired-end (PE) reads. Raw reads were filtered and trimmed using Trimmomatic (http://www.usadellab.org/cms/index.php?page=trimmomatic) following four criteria, including reads with adaptors, reads with a percentage of low-quality bases (< 20) > 50%, reads with > 10% “N” bases, and trimmed reads shorter than 75 bp. All clean reads were mapped to the full-length reference transcriptome obtained from PacBio Iso-seq using bowtie 2 (v2.1.0). We used the FPMK (Fragments Per Kilobases per Million reads) method to estimate gene transcript abundance, which was obtained with default parameters in the RSEM (v1.2.6) Program^60^. The DESeq2 Package^61^ was used to identify DEGs between non-germinated and germinated seeds. Significant DEGs were filtered with a false discovery rate (FDR) < 0.005 and a minimum two-fold change. GO enrichment analysis of DEGs was performed with the Goseq Package and GO terms with p-values < 0.05 were considered significantly enriched. KEGG pathway enrichment analysis was performed with KOBAS (v2.0) Software based on a corrected p value of 0.05^34^.

### Small RNA sequencing and data analysis pipeline

Seeds from three biological replicates were sampled, and only RNA samples from the first (NGS, day 0) and last (GS, day 90) time points were used for small RNA-seq. Small RNAs from total RNAs were isolated with agarose gel electrophoresis by selecting 18–30 nt fragments. A total of six small RNA-seq libraries was constructed using the NEBNext Multiplex Small RNA Library Prep Set following the manufacturer’s instructions. Libraries were then sequenced using the Illumina Hi-seq System to generate 50 bp single-end (PE) reads. Raw reads were filtered and trimmed using the Trimmomatic Program (v0.30; http://www.usadellab.org/cms/index.php?page=trimmomatic) and trimmed reads shorter than 18 bp were filtered out. The clean RNA reads were blasted to the Rfam database (http://rfam.xfam.org) to identify and remove tRNAs, rRNAs, scRNAs, and snRNAs with a cut-off E-value of 1e−5. To identify known miRNAs, the clean reads were first mapped to the reference transcriptome to remove aligned sequences. The unmapped reads were then matched to MiRBase (http://www.mirbase.org/) and the annotated miRNAs were considered as known miRNAs. Novel miRNAs were predicted using miRDeep2 Software^62^. The expression of miRNAs was estimated by transcripts per million (TPM), and miRNAs with fold changes > 2 and p-values < 0.05 were identified as differentially expressed miRNAs. TargetFinder Software (https://github.com/carringtonlab/TargetFinder) with default parameters was used to predict the target genes of miRNAs. GO and KEGG enrichment analyses of miRNA target genes were performed as described for the mRNA sequencing and data analysis pipeline. The figure was constructed by TBtools 1.046 (https://github.com/CJ-Chen/TBtools)63.

### Measurements of ABA and GA_3_ contents in *M. sieboldii* seeds

Endogenous ABA and GA_3_ levels were determined by high-performance liquid chromatography (HPLC)^64^. Briefly, frozen seed samples were transferred to a mortar and ground into powder, which was then homogenized and extracted for 24 h in 80% methanol with an internal standard. The purification was carried out with an Oasis Max solid phase extract cartridge and eluted with 5% formic acid in methanol. Subsequently, the eluted sample was dried, reconstituted, and injected into a liquid chromatography–tandem mass spectrometry system consisting of an Acquity ultra-performance liquid chromatograph^4^. Three biological replications were performed for non-germinated and germinated seeds.

### Ethical Statements

*M. sieboldii* seeds were used in this study. *M. sieboldii* is a National level 3 protection plants of China and the identification information is stored in the Plant Photo Bank of China (PPBC ID: 2657292). The seed sample was kindly provided by Benxi Botanical Garden (Benxi, Liaoning, China). We complied with all the relevant institutional, national and international guidelines.

## Supplementary Information

Supplementary Table S1.

Supplementary Figures.

## Data Availability

The sequence data of 14 samples reported in this study have been deposited into the NCBI Sequence Read Archive (SRA) database with the BioProject No. of PRJNA545617 (the accession https://www.ncbi.nlm.nih.gov/bioproject/PRJNA545617).
