# Supplementary material for: Integrated RNA and miRNA sequencing analysis reveals a complex regulatory network of Magnolia sieboldii seed germination
Source: Sci Rep. 2021 May 25;11:10842. doi: 10.1038/s41598-021-90270-y (PMC8149418; doi:10.1038/s41598-021-90270-y)
Supplement: Supplementary file 1 — Supplementary Table S1. [file 41598_2021_90270_MOESM1_ESM.pdf]

Integrated RNA and miRNA sequencing analysis reveals a complex regulatory network of *Magnolia sieboldii* seed germination

Mei Mei [1], Jun Wei [2], Wanfeng Ai [1], Lijie Zhang [3] and Xiu-jun Lu [3,*]
[1]Department of Horticulture, Shenyang Agricultural University, Shenyang, China
[2]Institute of Botany, Chinese Academy of Sciences, Beijing, China
[3]Department of Forestry, Shenyang Agricultural University, Shenyang, China

Supplementary Table S1. The negative correlaiton of gene expression between miRNA and their targeted mRNA.

mRNA_ID	mRNA_logFC	miRNA_ID	miRNA_logFC
PB.316591.1	-9.25	gma-miR319q	1.01
PB.158142.1	-8.04	bdi-miR529-5p	3.04
PB.240299.1	-4.12	ppt-miR529f	1.78
PB.323819.1	-8.54	pta-miR159a	1.80
PB.312787.1	5.98	Novel3874	-4.47
PB.278794.1	-4.25	Novel6504	1.01
PB.278794.1	-4.25	Novel7890	1.80
PB.230486.1	5.42	aqc-miR482c	-1.93
PB.230486.1	5.42	pab-miR11534	-6.91
PB.312500.1	-3.48	Novel6504	1.01
PB.312500.1	-3.48	Novel7890	1.80
PB.312500.1	-3.48	Novel9132	1.62
PB.67424.1	-8.47	bdi-miR529-5p	3.04
PB.159652.1	-1.94	Novel6504	1.01
PB.159652.1	-1.94	Novel7890	1.80
PB.1373.1	-5.20	csi-miR3951a-5p	1.25
PB.202168.3	-4.85	bdi-miR529-5p	3.04
PB.210069.1	-4.06	cpo-miR-203-3p	1.16
PB.173950.1	-5.39	ppt-miR529f	1.78
PB.202168.3	-4.85	ppt-miR529f	1.78
PB.292330.1	2.42	Novel3874	-4.47
PB.160653.1	-3.79	bdi-miR159a-3p	3.65
PB.160653.1	-3.79	gma-miR319l	1.54
PB.160653.1	-3.79	pab-miR159c	2.75
PB.160653.1	-3.79	pta-miR159a	1.80
PB.160653.1	-3.79	smo-miR159	2.21
PB.224523.1	-10.56	gma-miR319q	1.01
PB.307179.1	-7.19	gma-miR319q	1.01
PB.87836.1	-7.45	gma-miR319q	1.01
PB.95131.1	-5.57	csi-miR3951a-5p	1.25
PB.55702.1	-1.41	atr-miR171b	1.05
PB.55702.1	-1.41	mtr-miR171c	1.02
PB.55702.1	-1.41	pab-miR396a-5p	1.26
PB.55702.1	-1.41	sly-miR171e	1.62
PB.55702.1	-1.41	smo-miR171a	5.59
PB.169439.1	1.52	Novel11431	-1.32
PB.169439.1	1.52	Novel11720	-1.69
PB.169439.1	1.52	Novel12011	-4.91
PB.169439.1	1.52	Novel8804	-1.10
PB.169439.1	1.52	Novel9223	-4.63
PB.178018.1	-2.75	csi-miR396f-5p	2.04

PB.181926.1	-4.15	csi-miR396f-5p	2.04
PB.307475.1	-6.59	csi-miR396f-5p	2.04
PB.181926.1	-4.15	pab-miR396a-5p	1.26
PB.307475.1	-6.59	pab-miR396a-5p	1.26
PB.184987.3	-7.26	csi-miR396f-5p	2.04
PB.184987.3	-7.26	pab-miR396a-5p	1.26
PB.307390.1	-8.67	gma-miR319q	1.01
PB.281282.1	-2.12	bdi-miR159a-3p	3.65
PB.317174.1	-9.51	gma-miR319q	1.01
PB.317174.1	-9.51	csi-miR3951a-5p	1.25
PB.197222.1	-5.31	gma-miR319q	1.01
PB.197222.1	-5.31	Novel6504	1.01
PB.197222.1	-5.31	Novel7890	1.80
PB.144628.1	-10.21	gma-miR319l	1.54
PB.144628.1	-10.21	gma-miR319q	1.01
PB.215339.1	1.18	Novel11431	-1.32
PB.215339.1	1.18	Novel11720	-1.69
PB.215339.1	1.18	Novel12011	-4.91
PB.215339.1	1.18	Novel8804	-1.10
PB.215339.1	1.18	Novel9223	-4.63
PB.108231.1	-1.84	pta-miR159a	1.80
PB.119961.1	4.16	Novel11431	-1.32
PB.119961.1	4.16	Novel11720	-1.69
PB.119961.1	4.16	Novel12011	-4.91
PB.119961.1	4.16	Novel8804	-1.10
PB.150627.1	-1.62	Novel7890	1.80
PB.159466.1	-1.28	gma-miR319q	1.01
PB.174380.1	-1.81	pab-miR159c	2.75
PB.214317.1	-6.42	csi-miR3951a-5p	1.25
PB.23114.1	-6.63	bdi-miR529-5p	3.04
PB.23114.1	-6.63	ppt-miR529f	1.78
PB.292378.1	5.68	Novel3874	-4.47
PB.307752.1	6.19	pab-miR11534	-6.91
PB.313728.1	1.63	Novel3874	-4.47
PB.326611.1	8.27	Novel5220	-4.47
PB.326611.1	8.27	Novel8299	-4.68
PB.259836.1	8.69	Novel3874	-4.47
PB.316643.1	3.57	pab-miR11534	-6.91
PB.37024.1	3.13	pab-miR11534	-6.91
PB.270495.1	-3.25	csi-miR3951a-5p	1.25
PB.223676.1	-2.97	csi-miR3951a-5p	1.25
PB.221216.1	3.34	pab-miR11534	-6.91
PB.208087.1	2.92	pab-miR11534	-6.91
PB.208087.1	2.92	pab-miR11534	-6.91

PB.303896.1	-3.71	bdi-miR529-5p	3.04
PB.177012.1	-5.64	pab-miR159c	2.75
PB.177012.1	-5.64	pta-miR159a	1.80
PB.305517.1	-11.35	pab-miR159c	2.75
PB.305517.1	-11.35	smo-miR159	2.21
PB.188390.1	-4.98	csi-miR396f-5p	2.04
PB.326908.1	-7.68	pta-miR159a	1.80
PB.305517.1	-11.35	gma-miR319q	1.01
PB.315508.1	-8.88	gma-miR319q	1.01
PB.257297.1	-3.94	gma-miR319q	1.01
PB.117276.1	5.93	pab-miR11534	-6.91
