# Supplementary material for: Integrated RNA and miRNA sequencing analysis reveals a complex regulatory network of Magnolia sieboldii seed germination
Source: Sci Rep. 2021 May 25;11:10842. doi: 10.1038/s41598-021-90270-y (PMC8149418; doi:10.1038/s41598-021-90270-y)
Supplement: Supplementary file 2 — Supplementary Figures. [file 41598_2021_90270_MOESM2_ESM.pdf]

Integrated RNA and miRNA sequencing analysis reveals a complex regulatory network of *Magnolia sieboldii* seed germination

Mei Mei [1], Jun Wei [2], Wanfeng Ai [1], Lijie Zhang [3] and Xiu-jun Lu [3,*]
[1]Department of Horticulture, Shenyang Agricultural University, Shenyang, China
[2]Institute of Botany, Chinese Academy of Sciences, Beijing, China
[3]Department of Forestry, Shenyang Agricultural University, Shenyang, China
*Correspondence: lxjsyau@syau.edu.cn (X. Lu)

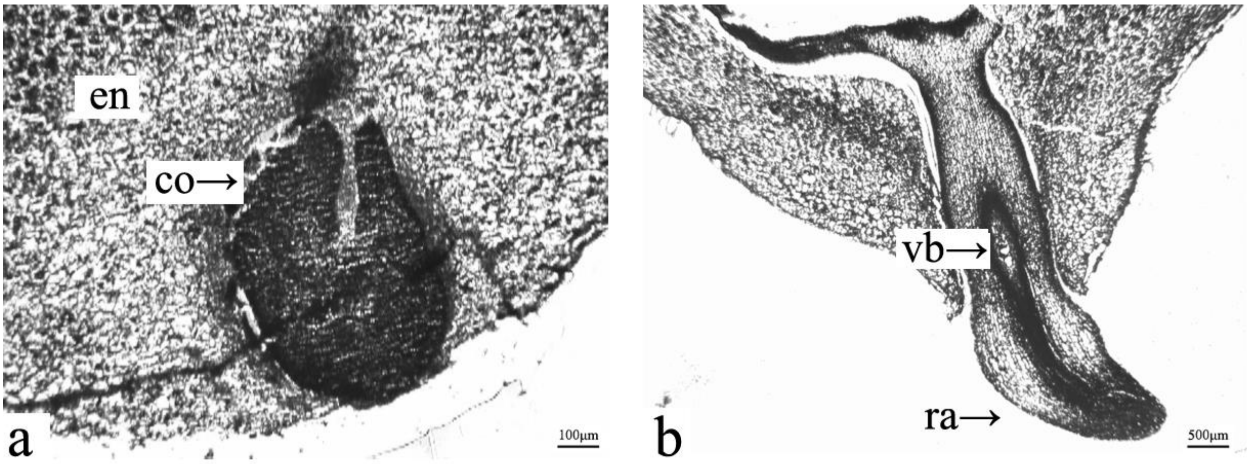

Supplementary Figure S1. Comparison between non−germinated and germinated seeds. (a) non−germinated seed, en: endosperm; co: cotyledon. (b) germinated seed, vb: vascular bundle ra: radicle.

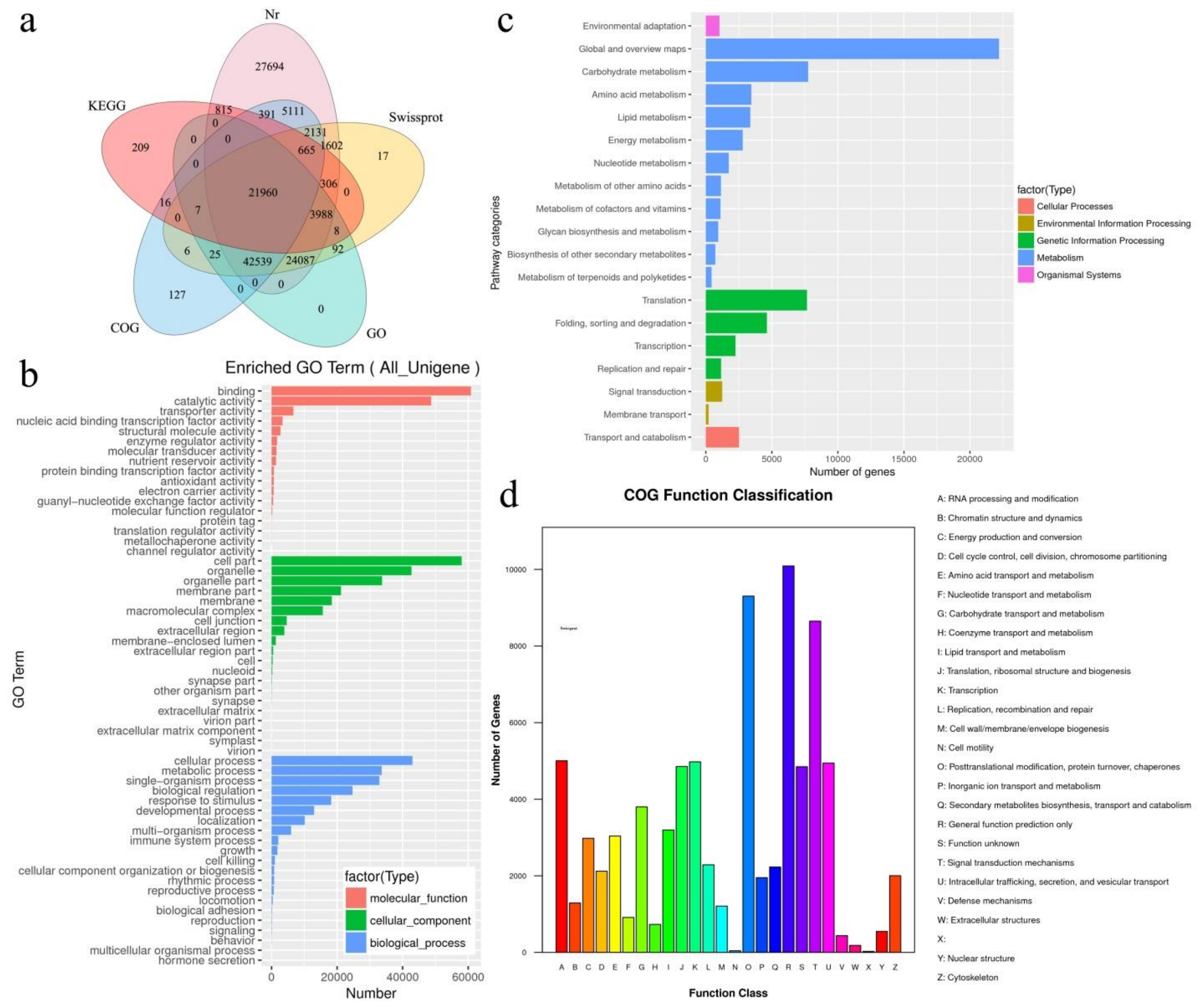

Supplementary Figure S2. Functional annotation of the full-length reference transcriptome. (a) Venn diagram of annotated transcripts, which matched in the non-redundant proteins (Nr) database, the SwissProt database (Swissprot), Kyoto Encyclopedia Genes and Genomes (KEGG), and the Clusters of Orthologous Groups of proteins (COG). (b) Gene ontology classification of the reference transcriptome. (c) KEGG classification of the reference transcriptome. (d) COG classification of the reference transcriptome

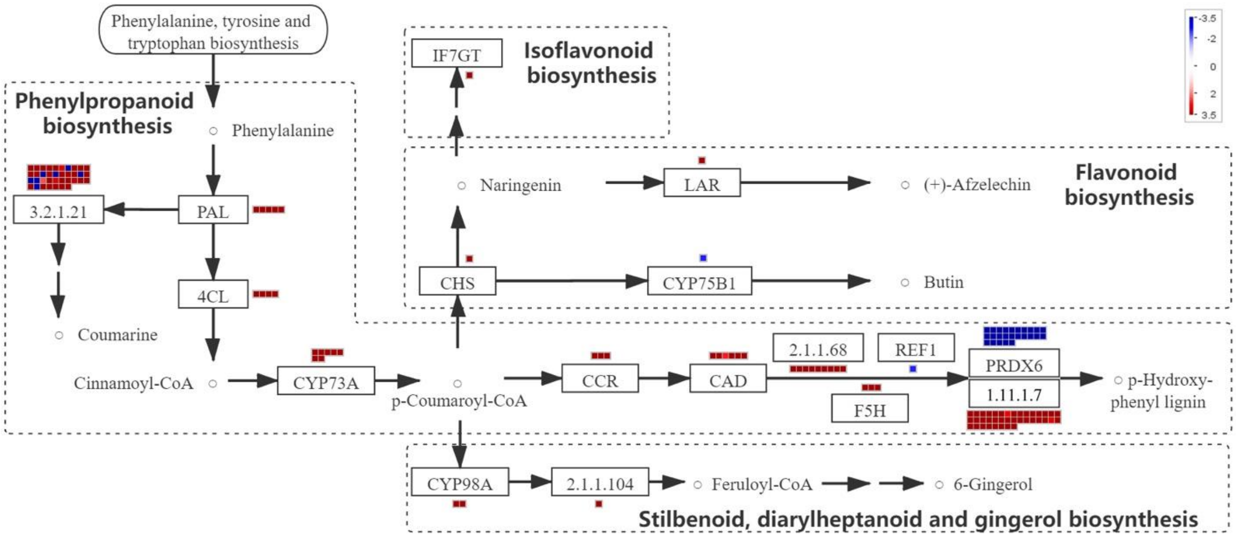

Supplementary Figure S3. Regulatory network of major secondary metabolic processes. Red and blue rectangles indicate up-regulated and down-regulated genes, respectively.
